# Similar rates of morphological evolution in domesticated and wild pigs and dogs

**DOI:** 10.1186/s12983-018-0265-x

**Published:** 2018-05-23

**Authors:** Madeleine Geiger, Marcelo R. Sánchez-Villagra

**Affiliations:** 1 Universität Zürich, Paläontologisches Institut und Museum, Karl-Schmid-Strasse 4, 8006 Zürich, Switzerland; 2 Department of Zoology, University of Cambridge, Downing Street, Cambridge, CB2 3EJ UK

**Keywords:** Evolutionary rate, Darwins, Haldanes, Domestication, Mammals, *Canis*, *Sus*

## Abstract

**Background:**

Whether the great morphological disparity of domesticated forms is the result of uniformly higher evolutionary rates compared to the wild populations is debated. We provide new data on changes of skull dimensions within historical time periods in wild and domesticated dogs and pigs to test if domestication might lead to an accelerated tempo of evolution in comparison to the wild conspecifics. Darwins and Haldanes were used to quantify evolutionary rates. Comparisons with evolutionary rates in other species and concerning other characteristics from the literature were conducted.

**Results:**

Newly gathered and literature data show that most skull dimensions do not change faster in domesticated breeds than in wild populations, although it is well known that there is extensive artificial selection on skull shape in some dog breeds. Evolutionary rates among domesticated forms and traits (e.g., production traits in pigs, and racing speed in some horses and greyhounds) might vary greatly with species and breeding aim.

**Conclusions:**

Our study shows that evolutionary rates in domestication are not in any event faster than those in the wild, although they are often perceived as such given the vast changes that appear in a relatively short period of time. This may imply that evolution under natural conditions – i.e., without human intervention – is not as slow as previously described, for example by Darwin. On the other hand, our results illustrate how diverse domestication is in tempo, mode, and processes involved.

**Electronic supplementary material:**

The online version of this article (10.1186/s12983-018-0265-x) contains supplementary material, which is available to authorized users.

## Background

The impressive diversity in domesticated forms, attained in historical times [1], has led to the claim that rates of evolution, i.e., the tempo or pace of evolutionary change, in domesticated populations is higher than in wild populations [2]. As opposed to this view, it has been found that evolutionary rates during the domestication of some plants were lower, or at least similar in speed, as those of natural populations [3]. Comparative studies on evolutionary rates in wild vs. domesticated species are scarce. We provide new data to address this issue using dogs and pigs as case studies. Domestic dogs and pigs exhibit a much greater amount of morphological disparity than their wild conspecifics, as reflected for example in skull proportions of Yorkshire pig vs. potbellied pig or Irish wolfhound vs. pug [4–6]. Other domesticates, on the other hand, are much less morphologically diverse, such as for example domesticated horses and cats [6, 7]. Great differences in disparity of skull shape between wild and domestic form provide a basis for the quantification and comparison of the rate of change over time in the domestic vs. the wild state.

In domestic dogs and pigs, the considerable morphological disparity occurred in what is perceived as a relatively short period of time [1]. Dogs were domesticated from wolves (*Canis lupus*) between ~ 14′000 and 40′000 years before present ([8, 9], for a review of earlier studies see [10]) and already in relatively early phases of domestication, different types of dogs could be distinguished [5]. Since the formation of kennel clubs and breeding standards about 150 years ago, more than 300 different breeds have been defined [11]. Domestic pigs have been domesticated later than dogs, at least 8000 years before present, from wild boar (*Sus scrofa*) [12, 13]. Dozens of different breeds exist today, mainly bred for efficient pork production and reproduction [4, 14].

Many domestic animal and plant varieties have been selected for better performance, e.g., oil and protein content in corn [15], adult weight and growth rate in broiler chicken [16, 17], egg production in turkey [18], milk yield in dairy cattle [19], and racing speed in horses [20], to only name a few. Artificial selection for the “improvement” of domestic species, however, is not limited to livestock and crops but can also affect aesthetic traits of domestic pets that are not directly linked to their performance. Some breeds of domestic dogs and pigs show a remarkable extent of morphological change over historical time periods. Examples are the St. Bernard dog [21–24], bullterrier [23], Newfoundland dog [25], and Berkshire pig [4, 26, 27]. All are highly specialized breeds which have been under intensive selection regimes during the last decades, either for appearance or performance. Since the eighteenth century, dogs were known from the St. Bernard hospice, Switzerland [24]. These medium sized, relatively lightweight dogs were bred as working and guarding dogs and were highly variable in outer appearance [24]. From the late nineteenth century onwards, there was a trend towards breeding increasingly heavier and bulldog-like St. Bernards with heavy heads, a pronounced angle between the snout and the frontal (“stop”), and a short snout – characteristics which are nowadays typical for this breed [24]. Similar trends have been described in the Newfoundland, although the trend towards a more dome shaped skull is not found in all breeding lines [25]. In the bullterrier, the angle between the rostrum and the cranial base (prebasial angle) has changed markedly throughout the twentieth century and more recent specimens of this breed tend to have a ventrally rotated rostrum and therefore an “egg” shaped skull [23]. In the Berkshire pig, morphology has changed several times during the history of this breed [4]. Berkshire pigs from a time period around 1935 (1933 - 1937), for example, were reported to exhibit a shorter, broader, and higher skull compared to pigs of the same breed from around  1900 (1882 - 1914) [26].

In this study, the quantitative changes in skull dimensions (linear measurements of the skull) in the above mentioned breeds of domestic dog and pig, which show a marked amount of skull shape change in historical time, and other groups to complement this biased selection (e.g. Siberian huskies that were probably selected for functional traits [28]), were investigated using time series that span the late 19th, entire 20th and early 21st centuries. These time series allowed for the estimation of evolutionary rates within one breed or population (darwin [2] and haldane [29] estimates), which quantify the proportional change of a trait (in this case skull dimensions) per million years or per generations that have passed between two points in time (for details see Methods). Estimated evolutionary rates of changes of skull dimensions were compared to those of distinct groups of the wild conspecifics of domestic dog and pig. Additionally, literature data on various evolutionary rates were compared in order to address the question if domesticates generally evolve slower, faster, or at an equal rate when compared to wild populations. We hypothesise that due to directed artificial selection by humans, skull dimensions and also other, e.g., physiological traits, in domestic forms evolve at higher rates compared to wild forms.

## Methods

Standard length, width, and height measurements of the skull (Table 1) were used to capture the overall skull shape and size and to calculate evolutionary rates (details below). We investigated specimens that stem from one breed and/or a similar and specified geographic area and have died in different years (see below and Table 2). We assumed that such populations in the wild and domestic breeds in one country or area of a country constitute a group of potentially interbreeding specimens and are thus comparable. Therefore, we could record changes of traits through time within one population or breed (allochronic study design [30]).

**Table 1:** Skull measurements used in this study and their definitions

Measurement	Description
Skull base length	Distance between the rostral border of the foramen magnum to the junction between pterygoid, palatinum, and praesphenoid, measured on the midline between left and right sides.
Skull length^a^	Distance between the tip of the premaxilla, at the level of the alveoli of the incisive teeth, to the rostral border of the foramen magnum.
Zygomatic breadth	Maximum breadth of the zygomatic arches.
Palatal length^b^	Distance between the tip of the premaxilla, at the level of the alveoli of the incisive teeth, to the most distal point of the palatine torus, measured at the interpalatine suture.
Palatal breadth	Maximum breadth of the palate, measured at the internal margins of the left and right upper tooth rows between P4 and M1 at the level of the alveoli.
Skull height^c^	Vertical distance between the rostral border of the foramen magnum to the most superior margin of the skull (at the occipital, intraparietal, or parietal bones; including the sagittal crest, if present).
Nasal length	Distance between the junction of the nasal and frontal bones to the most distal tip of the nasals, measured at the internasal suture.
Prebasial angle	Angle between the cranial base (skull base length) and the hard palate (distance between the most caudal point of the palatal bones at the interpalatine suture to the median point of a virtual line connecting the most caudal points of the two palatine fissures, on the plane of the palatal bone).

^a^In St. Bernard, Newfoundland, bullterrier, Siberian husky, and wolf, this measurement was taken at the anterior side of the incisive teeth; in the other domestic dog breeds (boxer, barsoi, dachshund, dogue de Bordeaux, greyhound, French bulldog, Bernese mountain dog, and Chihuahua) this measurement was taken at the posterior (caudal) side of the incisive teeth. In the wild boar, this measurement was taken at the anterior side of the incisive teeth; in the Berkshire pig, this measurement was described as “von der Spitze der Zwischenkiefer zum unteren Rand des Foramen magnum” [26, 27], i.e., from the tip of the interpremaxillary suture to the lower margin of the foramen magnum

^b^In the domestic dogs and the wolf, this measurement was taken at the posterior side of the incisive teeth. In the wild boar, this measurement was taken at the anterior side of the incisive teeth. In the Berkshire pig, this measurement was described as “von der Spitze der Zwischenkiefer zum Gaumenausschnitt” [26, 27], i.e., from the tip of the interpremaxillary suture to the posterior naris

^c^in the Berkshire pig, this measurement was described as “vom Foramen Magnum zur Mitte des Occipitalkammes” [26, 27], i.e., from the foramen magnum to the midpoint of the occipital crest

**Table 2:** Investigated time series of domestic dog, domestic pig, wolf, and wild boar skulls

Groups	n	Investigated time period	Number of years	Number of generations	References
Domestic dogs					
St. Bernard	72	1885-2012	127	103.3	This study
Newfoundland	8	1899-1996	97	78.9	This study
Boxer	13	1928-2004	76	61.8	[32]
Barsoi	9	1937-2010	73	59.3	[32]
Bullterrier	17	1932-2000	68	55.3	This study
Dachshund	15	1931-1997	66	53.7	[32]
French bulldog	17	1933-1995	62	50.4	[32]
Greyhound	18	1932-1986	54	43.9	[32]
Dogue de Bordeaux	7	1955-1998	43	35	[32]
Siberian husky	8	1885-1926	41	33	This study
Bernese mountain dog	16	1970-1998	28	22.8	[32]
Chihuahua	30	1987-2009	22	17.9	[32]
Wild form of domestic dogs					
Wolf	27	1959-2015	56	28	This study
Domestic pig					
Berkshire pig	8	1896-1936	40	52	[26, 27]
Wild form of domestic pigs					
Wild boar	15	1873-1999	126	63	This study

N, number of investigated specimens. Breeds of domestic dogs are ordered according to length of investigated time period, from longest to shortest

The time periods over which phenotypic changes were investigated in this study were similar among groups, i.e., breeds and populations (within about one century, Table 2) and thus prevented possible biases of the evolutionary rate estimates by differing time periods [30, 31]. Time series in every group were composed of specimens of similar age stage, i.e., similar absolute or dental age, to ensure that ontogenetic variation does not bias the evolutionary rate estimates. Where possible, care was taken to sample specimens equally distributed over the considered time period of that group (e.g. equal amount of specimens at the beginning and the end of the collection period), but this was not always possible. Further, not the same time period could be considered in all groups (e.g., some groups represent the first half of the twentieth century, others the second half). Both sexes were included. Not all measurements could be taken in all specimens and all groups (see below). The measurements for each group were either taken by the same person (Table 2; this study), or the persons who contributed measurements were working in close collaboration with one another (Table 2; this study and [32]; [26] and [27]). Therefore, no sampling bias is to be expected. The investigated skulls are housed in museum collections and thus no specimen was killed for this study and no live specimens were used. Specimens are housed in the collections of the Albert-Heim-Stiftung at the Naturhistorisches Museum Bern, Switzerland, the Zoological Institute of the Russian Academy of Science, Sankt-Peterburg, Russia, and the Museum für Naturkunde, Berlin, Germany. Raw measurements are available in Additional file 1.

For comparisons we collected published data on evolutionary rates across different traits and species within a timeframe of maximum 300 years and considered also studies that report on single traits that have been selected in different domesticated species. Only evolutionary rates from allochronic study designs (reporting rates of evolution within one line, not divergence) and based on phenotypic data (not genetic) were used for these comparisons [30].

### Domestic dogs and wolves

Four domestic dog groups (St. Bernard, Newfoundland, bullterrier, and Siberian husky) and wolves were measured in this study (Table 2). The first three domestic dog groups were chosen because their skull shape is known to have changed markedly throughout the last 150 years (see Background). Our sample was extended with data on eight domestic dog groups (boxer, barsoi, dogue de Bordeaux. greyhound, French bulldog, Bernese mountain dog, and Chihuahua) to extend this biased sampling, based on specimens studied and measured by Geiger and Haussman [32] using similar measurement methods. Only dentally mature specimens, i.e., with all permanent teeth erupted into occlusion, were used.

In the St. Bernard, Newfoundland, bullterrier, Siberian husky, and the wolf seven linear measurements were taken with calipers (Table 1). Additionally, the prebasial angle (Table 1) was measured in St. Bernards and bullterriers, because there is evidence that these breeds show substantial change of this trait over time [21, 23]. The prebasial angle was measured as described in Nussbaumer [23] and Baxter and Nussbaumer [33] using a contour gauge. In the second group, which comprises the data by Geiger and Haussman [32], three linear measurements were taken with callipers: skull base length, skull length, and zygomatic breadth (Table 1).

Most studied skulls (except the Siberian huskies) are from purebred individuals for which pedigree information and origin are known and which represent modern breeds with breeding standards [21, 23]. All those specimens are from Swiss kennels (some specimens stem from the same kennel). The here used Siberian huskies represent a local sledge dog population from the region of Sakha (Yakutia) in Russia and died between 1885 and 1926 [28]. They are therefore representatives of a group of dogs that lived in a time before the Siberian huskies were recognized as a breed by the American Kennel Club in the United States of America in 1930 [34] and were probably selected for functional traits [28]. The here investigated wolf specimens stem from the Leningrad Oblast, a region of Russia that includes the city of Sankt-Peterburg and encompasses about 84′000 km^2^.

### Domestic pigs and wild boar

The studied wild boar specimens lived in an area within about 200 km around the city of Berlin, Germany. Only dentally mature wild boars, i.e., specimens with all permanent teeth fully erupted into occlusion, were used. We quantified changes in skull measurements on specimens of the Berkshire pig based on raw data provided by two different but related studies [26, 27] (Table 2). Data of Berkshire pig specimens between about two and three years of age were used. Different investigated age stages in wild boar and Berkshire pig were not supposed to produce a bias because the age stages within each group were similar.

### Analyses

Evolutionary rates were calculated in darwins [2] and haldanes. [29] (for a review see [30]). Since we used continuous time series evolutionary rates were estimated using univariate least-squares regressions as described by Hendry and Kinnison [30] and Purugganan and Fuller [3]. For every measurement and every group (Table 2), regressions were computed with the natural logarithm of the measurements (darwin estimates) and the natural logarithm of the measurements divided by the standard deviation (haldane estimates) as the dependent variables. The time in million years (darwin estimates) and the number of generations (haldane estimates) were used as independent variables [3, 30]. These methods operate on the assumption that the evolutionary rates are linear and not changing over the investigated time period, which we could not test here. Since different variables (skull dimensions) were assessed from the same sample, the *p*-values were Bonferroni-corrected by dividing the significance level (a = 0.05) by the number of tests conducted in each group (e.g., in the St. Bernard, where eight variables were assessed, a = 0.05 was divided by eight).

Generation time was calculated as the species mean age at attainment of sexual maturity plus gestation length, adjusted for seasonality (Table 3). These values of life history variables were obtained from the literature; for domestic dogs, mean sexual maturity across breeds was calculated. Most wild mammals are seasonal breeders but many domestic forms breed non-seasonally (e.g., [4]). Therefore, the wild forms are unlikely to breed in between the breading seasons and their generation time is subsequently given in whole years, as opposed to fractions of years in domesticates. Our method contrasts with many other studies that often use only age at maturity as an estimate for generation time [30]. However, we argue that gestation time and seasonality are equally important components of generation time as sexual maturity and can add a substantial amount of time to the generation time estimate of a species. For example, gestation time is about one third of the age at sexual maturity in wild boar and additionally, the generation time has to be rounded up from 15.5 to 24 months because wild boar are unlikely to breed in between seasons, i.e., between 12 and 24 months of age (Table 3). In wolves, in which a generation time of 24.1 months was calculated here (22 months until sexual maturity plus 2.1 months gestation time), a generation time of 24 months, i.e., 2 years, was assumed (Table 3).

**Table 3:** Estimates of generation time in the here investigated groups

Species	Sexual maturity (months)	References for sexual maturity	Gestation time (months)	References for gestation time	Seasonality	Generation time (months)
Domestic dog	12.5	[61]	2.2	[62]	no	14.7
Wolf	22.0	[63]	2.1	[64]	yes	24
Domestic pig	5.5	[65]	3.8	[66]	no	9.3
Wild boar	11.7	[67]	3.8	[67]	yes	24
Domestic horse	13.5	[68]	11.4	[69]	no	24.9

Generation time is the sum of the age at sexual maturity and gestation time, rounded to the next full year in groups with seasonal reproduction

The slope of the regressions were used as evolutionary rate estimates. The same computation of rates was used for those references that did not provide rate estimates, but raw data only. The sign of a slope (plus or minus) shows if a measurement tends to get smaller or larger over time, but for the interpretation of the magnitude of change only absolute values were used. For further analyses, mean and median evolutionary rates were calculated for different groups (because the skull measurements are not independent of each other, see Discussion). For the evaluation of similarity/difference of rate estimates between groups, non-parametric Wilcoxon rank sum tests and Wilcoxon signed rank test were conducted because of the relative small size of compared samples. All analyses that are reported here were performed using Microsoft Excel 2010, R version 3.4.0 [35], and RStudio version 1.0.143 [36].

## Results

### Evolutionary rate estimates

**Fig. 1:**
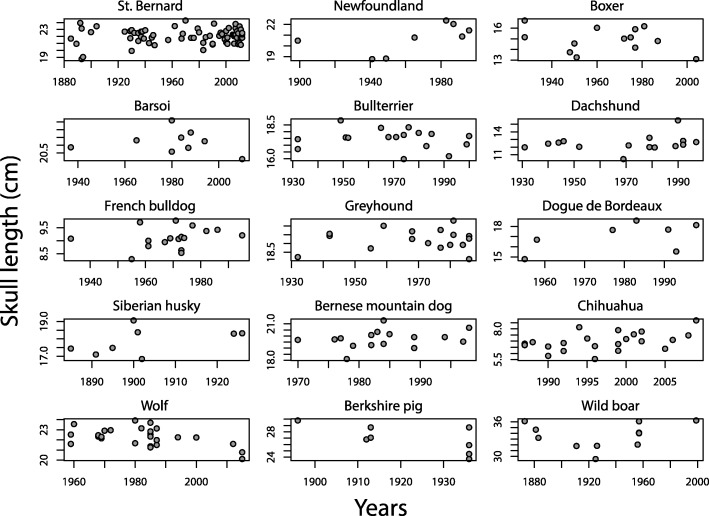
Historical change of maximum skull length in all of the here investigated groups. These plots illustrate the distribution of sampled specimens over the investigated time period in every group

**Fig. 2:**
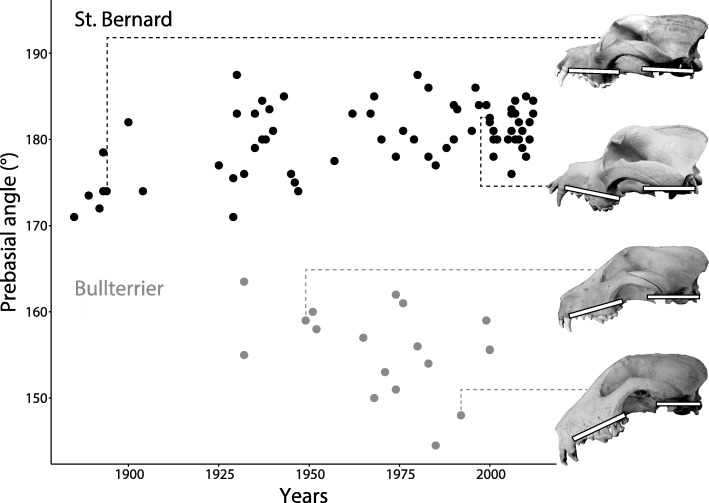
Historical change in one of the here investigated skull variables in two domestic dog breeds. Although a change of the prebasial angle (angle between the hard palate and the cranial base of the skull) throughout many decades can be discerned in both breeds (dorsal bending in the St. Bernard and ventral bending in the bullterrier), a significant change of this variable could only be found in the St. Bernard (see Tables 4 and 5). The prebasial angles are indicated with white bars on the depicted photographs of skulls, which are scaled to the same neurocranial length

Distributions of specimens over the investigated time periods in every group are shown in Fig. 1, based on the example of skull length. Among the domestic dog groups, only few measurements varied significantly with time, and these measurements were found in the St. Bernard dog (Tables 4 and 5): there is evidence for an increasing palatal breadth (F_1,70_ = 9.151, *p* = 0.003) and skull height (F_1,70_ = 11.09, *p* = 0.001) and a dorsal rotation of the rostrum relative to the cranial base (F_1,70_ = 23.89, *p* < 0.001) over the investigated time period. No significant changes of any of the investigated skull measurements over time were found in the bullterrier and Newfoundland, where changes were expected (see Background). Further, darwin and haldane estimates in these breeds were in a similar range as in the Siberian husky (Tables 4 and 5), where selection for skull shape was probably not as strong (see Background and Methods). Figure 2 illustrates historical change in one of the here tested variables, the prebasial angle, in two breeds: the St. Bernard, for which we could demonstrate a significant change and the bullterrier, in which we found no significant change (Tables 4 and 5).

**Table 4:** Estimated evolutionary rates of skull dimensions in darwins

	Skull base length	Skull length	Zygomatic breadth	Palatal length	Palatal breadth	Skull height	Nasal length	Prebasial angle
Domestic dogs								
St. Bernard	301 (0.043)	129 (0.011)	255 (0.030)	−10 (< 0.001)	472 (0.116)*	663 (0.137)*	− 799 (0.158)	276 (0.255)*
Newfoundland	504 (0.102)	1126 (0.322)	1263 (0.339)	1245 (0.364)	660 (0.069)	956 (0.206)	–	–
Boxer	63 (< 0.001)	− 877 (0.067)	− 142 (0.004)	–	–	–	–	–
Barsoi	− 1310 (0.212)	− 239 (0.020)	− 1298 (0.382)	–	–	–	–	–
Bullterrier	157 (0.004)	−310 (0.023)	− 505 (0.062)	186 (0.007)	− 544 (0.068)	−82 (0.003)	1443 (0.177)	− 695 (0.183)
Dachshund	583 (0.021)	870 (0.055)	− 295 (0.008)	–	–	–	–	–
French bulldog	535 (0.012)	808 (0.062)	896 (0.045)	–	–	–	–	–
Greyhound	−194 (0.017)	79 (0.002)	− 1070 (0.141)	–	–	–	–	–
Dogue de Bordeaux	2603 (0.276)	2565 (0.266)	2720 (0.299)	–	–	–	–	–
Siberian husky	1130 (0.162)	1329 (0.212)	665 (0.033)	1552 (0.290)	− 773 (0.032)	1471 (0.183)	−158 (0.001)	–
Bernese mountain dog	− 700 (0.007)	1266 (0.070)	− 1155 (0.039)	–	–	–	–	–
Chihuahua	1906 (0.013)	7056 (0.192)	5052 (0.149)	–	–	–	–	–
Wild form of domestic dogs								
Wolf	− 1349 (0.214)	− 1343 (0.262)*	− 850 (0.052)	− 1205 (0.172)	− 1680 (0.269)*	− 1045 (0.155)	− 635 (0.038)	–
Domestic pig								
Berkshire pig	–	− 3470 (0.460)	− 35 (0.0001)	− 4085 (0.365)	632 (0.013)	2531 (0.196)	− 3708 (0.186)	–
Wild form of domestic pigs								
Wild boar	18 (< 0.001)	231 (0.021)	127 (0.005)	96 (0.002)	259 (0.016)	630 (0.051)	388 (0.026)	–

Numbers in brackets are the r^2^ values of the regressions and asterisk and bold font indicate significant regressions (significance levels are Bonferroni corrected to account for multiple testing). Breeds of domestic dogs are ordered according to length of investigated time period, from longest to shortest (Table 2). For descriptions of skull dimensions see Table 1

**Table 5:** Estimated evolutionary rates of skull dimensions in haldanes

	Skull base length	Skull length	Zygomatic breadth	Palatal length	Palatal breadth	Skull height	Nasal length	Prebasial angle
Domestic dogs								
St. Bernard	0.007 (0.043)	0.003 (0.011)	0.006 (0.030)	−0.0002 (< 0.001)	0.011 (0.116)*	0.012 (0.137)*	−0.011 (0.158)	0.016 (0.255)*
Newfoundland	0.013 (0.102)	0.023 (0.322)	0.023 (0.339)	0.024 (0.364)	0.010 (0.069)	0.018 (0.206)	–	–
Boxer	0.001 (< 0.001)	−0.022 (0.067)	−0.004 (0.004)	–	–	–	–	–
Barsoi	−0.030 (0.212)	−0.009 (0.020)	− 0.040 (0.383)	–	–	–	–	–
Bullterrier	0.004 (0.004)	−0.009 (0.023)	−0.015 (0.062)	0.005 (0.007)	−0.016 (0.068)	− 0.003 (0.003)	0.025 (0.177)	− 0.026 (0.183)
Dachshund	0.008 (0.021)	0.014 (0.055)	−0.005 (0.008)	–	–	–	–	–
French bulldog	0.010 (0.012)	0.023 (0.062)	0.020 (0.045)	–	–	–	–	–
Greyhound	−0.009 (0.017)	0.003 (0.002)	−0.027 (0.141)	–	–	–	–	–
Dogue de Bordeaux	0.041 (0.276)	0.040 (0.266)	0.043 (0.299)	–	–	–	–	–
Siberian husky	0.035 (0.157)	0.041 (0.205)	0.016 (0.030)	0.048 (0.285)	−0.016 (0.034)	0.038 (0.177)	−0.004 (0.002)	–
Bernese mountain dog	−0.014 (0.007)	0.044 (0.070)	−0.032 (0.039)	–	–	–	–	–
Chihuahua	0.021 (0.013)	0.082 (0.192)	0.073 (0.149)	–	–	–	–	–
Wild form of domestic dogs								
Wolf	−0.061 (0.214)	−0.068 (0.262)*	− 0.030 (0.052)	−0.058 (0.172)	− 0.068 (0.269)*	−0.052 (0.155)	− 0.026 (0.038)	–
Domestic pig								
Berkshire pig	–	−0.036 (0.460)	−0.001 (< 0.001)	− 0.032 (0.365)	0.006 (0.013)	0.023 (0.200)	− 0.022 (0.186)	–
Wild form of domestic pigs								
Wild boar	0.0005 (< 0.001)	0.008 (0.021)	0.004 (0.005)	0.003 (0.002)	0.007 (0.016)	0.012 (0.051)	0.010 (0.026)	–

Numbers in brackets are the r^2^ values of the regressions and asterisk and bold font indicate significant regressions (significance levels are Bonferroni corrected to account for multiple testing). Breeds of domestic dogs are ordered according to length of investigated time period (years), from longest to shortest (Table 2). For descriptions of skull dimensions see Table 1

Significant changes of the examined skull variables with time were also found in the here investigated wolves (Tables 4 and 5): skull length (F_1,25_ = 8.871, *p* = 0.006) and palatal breadth (F_1,25_ = 9.202, p = 0.006). No significant changes of the examined skull measurements could be substantiated in the Berkshire pig and the investigated geographical group of wild boar (Tables 4 and 5).

### Comparisons domestic vs. wild

The median and mean evolutionary rates of the here investigated wild (boar, wolf) vs. domestic (pig, dog) groups are similar (darwins and haldanes, *p* > 0.5, Table 6). Furthermore, the median evolutionary rate of our domestic populations is similar to that of a large database of (micro-)evolutionary rates of different animals and traits regarding darwins but greater regarding haldanes (Table 6; [37]; Wilcoxon signed rank test, haldanes, V = 90, *p* = 0.002). Our evolutionary rate estimates for domestic populations were significantly smaller than the ones reported by Hendry et al. [38] for wild populations in an anthropogenic environment regarding darwins (populations that are impacted by humans; Wilcoxon rank sum test; darwins, W = 17, *p* < 0.001) and similar regarding haldanes. Further, our evolutionary rate estimates for domestic populations were similar regarding darwins or significantly smaller regarding haldanes than the rate estimates found by Hendry et al. [38] in undisturbed wild populations (Wilcoxon rank sum test; haldanes, W = 32, *p* = 0.005) (Table 6). In sum, we found no evidence for a faster rate of evolution of skull dimensions in our sample of domestic dogs and pigs relative to wild populations (regarding similar and different traits in related and unrelated wild forms).

**Table 6:** Summarised evolutionary rates in darwins and haldanes per investigated group and comparisons with the literature

Group	n	Max	Min	Median (or single value)	mean
Skull measurements					
Domestic dogs^a^	12	7056, 0.082	10, 0.0002	700, 0.016	1018, 0.021
Domestic pig (Berkshire pig)^a^	1	4085, 0.036	35, 0.001	3001, 0.023	2410, 0.020
Wolf (*Canis lupus*)^a^	1	1680, 0.068	635, 0.026	1205, 0.058	1158, 0.052
Wild boar (*Sus scrofa*)^a^	1	630, 0.012	18, 0.0005	231, 0.007	250, 0.006
Domestic (domestic dog and pig)^a^	13	7056, 0.082	10, 0.0002	773, 0.016	1159, 0.021
Wild (wolf and boar)^a^	2	1680, 0.068	18, 0.0005	633, 0.019	704, 0.029
Various traits					
Different clades^b^	30	–	–	1151, 0.006	–
Different clades, anthropogenic natural environment^c^	15, 7	38,931, 1.142	109, 0.003	8812, 0.145	12,430, 0.239
Different clades, undisturbed natural environment^c^	12, 14	11,171, 0.489	201, 0.003	2853, 0.102	3255, 0.161
Domestic pigs^d^	2	10,310, 0.032	4718, 0.029	7063, 0.032	7063, 0.031
Swedish standardbred trotter horses^e^	1	–	–	2065, 0.374	–
Greyhounds^f^	1	–	–	578, 0.039	–

Darwin estimates are given before the comma, haldanes after the comma. Max, maximum evolutionary rate; min, minimum evolutionary rate; n, number of groups (breeds, species, subgroups, systems) that have been included into this summary for the two rates estimates, respectively (Tables 4 and 5)

^a^this study, skull measurements

^b^Kinnison and Hendry [37], various phenotypic traits

^c^Hendry et al. [38], various phenotypic traits, allochronic data only

^d^Merks [14], production traits (weight gain (g/d), feed efficiency (kg/kg), and backfat thickness (mm)) in two pig breeds (Dutch landrace and great Yorkshire) from 1930 to 1990 (rates calculated in this study)

^e^Arnason [39], best average racing time (sec/km) in Swedish standardbred trotter horses from 1976 to 1994 (rates calculated in this study)

^f^Hill & Bünger [40] and Denny [41], raw data from [42], wintimes (sec) in the English Greyhound Derby (over 480 m) from 1929 to 2011 (rates calculated in this study)

Comparisons of our rate estimates with single traits of other domesticates from the literature revealed the following. The Swedish standardbred trotter horses got on average significantly faster (F_1,17_ = 562.4, *p* < 0.0001, r^2^ = 0.971) over 18 years at a rate of 2065 darwins and 0.374 haldanes (same regression results for haldanes and darwins; Table 6; [39]). This rate is significantly larger than the median of the domestic forms we studied (Wilcoxon signed rank test; darwins, V = 16, *p* = 0.040; haldanes, V = 0, *p* = 0.002) and similar (darwins) or significantly larger (haldanes) compared to the undisturbed wild population studied by Hendry et al. [38] (Wilcoxon signed rank test; haldanes, V = 5, *p* = 0.001; Table 6). Daily weight gain significantly increased and backfat thickness significantly decreased in Dutch landrace and great Yorkshire pigs (no changes in feed efficiency) over a period of 60 years at an average absolute rate of 7063 darwins and 0.031 haldanes (Table 6; Additional file 2: Table S1; [14]). This rate is significantly larger than (darwins) or similar to (haldanes) the one of the here investigated domestic forms (Wilcoxon signed rank test; darwins, V = 0, *p* < 0.001) and larger (darwins) as well as smaller (haldanes) than the undisturbed wild population studied by Hendry et al. [38] (Wilcoxon signed rank test; darwins, V = 4, *p* = 0.003; haldanes, V = 94, *p* = 0.007; Table 6). The winning time in the English Greyhound Derby decreased significantly (F_1,23_ = 72.13, p < 0.0001, r^2^ = 0.758) over 82 years at a rate of 578 darwins and 0.039 haldanes (F_1,23_ = 70.64, p < 0.0001, r^2^ = 0.754) (Table 6, [40, 41]; raw data from [42]) which means that the Greyhounds got significantly faster. This evolutionary rate is significantly smaller than the one which we found in our domestic group (Wilcoxon signed rank test; darwins, V = 75, p = 0.040; haldanes, V = 7, *p* = 0.008) and in the undisturbed wild populations by Hendry et al. [38] (Wilcoxon signed rank test; darwins, V = 74, p = 0.003; haldanes, V = 91, *p* = 0.013; Table 6). In sum, these comparisons show that different, selected traits in domesticates have the potential to evolve both faster and slower compared to wild populations.

## Discussion

There are different levels of human-animal/plant-interactions which may span the whole breadth from mere coexistence to full domestication and dependency [43, 44]. Apparently, these interaction-levels influence evolutionary rates differently: wild animals under anthropogenic disturbance and/or influence have been found to evolve faster than animals from undisturbed habitats [38]. In an investigation on traits in plants during the early domestication process, i.e., the transition between the wild and the domesticated state where interactions with humans are intensified, rates of evolution were reported as lower than, or comparable to, rates in wild populations [3]. These studies thus show that human intervention per se does not necessarily accelerate evolutionary rates of organisms [3, 38]. The current study further supports this conclusion.

Although evolutionary rates between wild and domestic forms appear to be similar (Table 6), we found evidence for marked differences among domestic groups and traits (Tables 4, 5 and 6), as has also been shown previously for improvement of production in livestock [45]. For some domestic dog breeds, it is well known that there have been changes of breeding standards or interpretations thereof and subsequent changes of skull shape and size were expected and could be confirmed with our data: the here reported skull shape changes in the St. Bernard corroborate the descriptions of changes given previously [21–24]. The skulls of the St. Bernard got more massive and heavy throughout the breeding history of the 19th and 20th centuries, with a more concave cranial vault, a more pronounced “stop”, and an increasing dorsal inclination of the rostrum (Fig. 2). We used partially the same sample as these studies, so this similarity of results is not surprising. However, not in all cases could reported skull dimension changes be quantitatively substantiated: (1) the bullterrier has been described to show a prominent decrease of the basicranial angle, i.e., a ventral rotation of the rostrum relative to the cranial base, throughout its breed history, although this could not been statistically confirmed [23]. We expanded the sample used by Nussbaumer [23] by incorporating more specimens until the year 2000 (Table 2), but we as well did not find a statistical significant decrease of the basicranial angle through time (Fig. 2, Tables 4 and 5), although a trend is clearly discernible; (2), in England, Newfoundlands have been selected for dome shaped skulls [25] but no changes related to higher and more convoluted skulls could be observed over the course of the last about 100 years in the investigated Newfoundlands from Switzerland (Tables 4 and 5). As indicated above, the aesthetic requirements were different in Switzerland and England, with Swiss standards being “more natural”, meaning less divergent [25]. In some domestic dogs, no marked changes were expected, as for example in the Siberian husky group that was probably not subject to artificial selection for aesthetic requirements; in this case in fact we did not find directed changes of skull dimensions (Tables 4 and 5). Selection for functional requirements might influence skull shape in these dogs, but there is no evidence for such a change in the sample at hand. Although the here observed inter-breed differences in the magnitude of changes over time might be the result of differential selection pressures, i.e., the absence or presence or the differing strength of directed artificial selection for a specific trait, the absence of a significant change of a trait over time might also be the result of differences in the examined time periods and differences in the selection pressures in these time periods (Fig. 1, Table 2), as well as relatively large variation of a trait within breeds (Fig. 2). Further, differences in relatedness among the examined domestic dog groups might have an influence on the observed changes or lack thereof. However, due to the reticulate nature of dog evolution, especially in modern breeds [46], predicting how relatedness of breeds among one another and the wolf influence evolutionary rates is difficult if not impossible.

The comparison of linear measurements has some limitations and would profit from complementation by advanced morphometric analysis, such as geometric morphometrics [47]. Such analyses have already been used for the investigation of craniodental shape variation in the domestication of dogs [21, 48–52] and pigs [53–55]. First, complex shape changes, e.g., dome shaped skull in Newfoundlands, cannot be captured by linear measurements. Second, the different linear skull dimensions are not independent from one another and parts of the skull function as non-independent, integrated modules [56, 57]. It is therefore to be expected that some of the linear dimensions of the skull change in a correlated fashion.

No significant changes of skull dimensions could be found in the Berkshire pigs, although such changes have been described previously [4, 26] (Tables 4 and 5). The evolutionary rates of the Berkshire pig lie within the range of domestic dogs (Tables 6). In contrast, comparatively fast evolutionary rates were reported for production traits, such as weight gain, in other domestic pig breeds [14] (Table 6). These contrasts reflect the history of pig breeding, in which performance, and not appearance, was and still is the prime concern of the breeders [4]. In contrast to the wild boar group that was examined in this study, for which no significant changes of skull traits could be shown over time, more change occurred in the here studied wolf group. The rate of morphological changes of the latter might reflect altered environmental conditions and/or selection pressures in the studied time period and geographical area.

## Conclusions

Our study shows that artificial selection in domesticated dogs and pigs does not necessarily result in evolutionary rates of skull dimensions significantly higher than those recorded in wild populations, although skull shape change over time has been described to be exceptionally pronounced in some dog and pig breeds. As evolutionary rates in domestication have been described as relatively fast compared to the rates under natural conditions [2], this would imply that evolution without human interventions can be faster than previously expected [58, 59]. (Or evolutionary rates in domestication are slower than previously expected.) However, our investigations show that evolutionary rates may vary greatly depending on species, breed, trait, breeding aim, evolutionary rate estimate (darwins or haldanes), generation time estimates, and the phase of domestication considered [60]. Further comparative studies on evolutionary rates in wild vs. domesticated species would therefore gain from investigating additional, also non-mammalian, species and different traits.

## Additional files

Additional file 1: Raw data on skull measurements. Each sheet contains collection identity numbers, sex and species (and breed/variety) affiliation, regional (if not a domestic breed) and temporal information, as well as all measurements of used specimen. (XLSX 48 kb)

Additional file 2: **Table S1.** Regression results of ln(trait value) vs. elapsed time in millions of years (to estimate darwins) and ln(trait value)/standard deviation vs. number of generations (to estimate haldanes), respectively, in two pig breeds. (DOCX 19 kb)
