# Supplementary material for: Similar rates of morphological evolution in domesticated and wild pigs and dogs
Source: Front Zool. 2018 May 23;15:23. doi: 10.1186/s12983-018-0265-x (PMC5966889; doi:10.1186/s12983-018-0265-x)
Supplement: Supplementary file 2 — Table S1. Regression results of ln(trait value) vs. elapsed time in millions of years (to estimate darwins) and ln(trait value)/standard deviation vs. number of generations (to estimate haldanes), respectively, in two pig breeds. (DOCX 19 kb) [file 12983_2018_265_MOESM2_ESM.docx]

**Table S1. Regression results of ln(trait value) vs. elapsed time in millions of years (to estimate darwins) and ln(trait value)/standard deviation vs. number of generations (to estimate haldanes), respectively, in two pig breeds** [1] **.** Df, degrees of freedom; p, p-value.

|  | **Daily gain** | | | | **Feed efficiency** | | | | **Backfat thickness** | | | |
| --- | --- | --- | --- | --- | --- | --- | --- | --- | --- | --- | --- | --- |
|  | **slope** | **F (df)** | **p** | **r^2^** | **slope** | **F (df)** | **p** | **r^2^** | **slope** | **F (df)** | **p** | **r^2^** |
| **Darwins** |  |  |  |  |  |  |  |  |  |  |  |  |
| **Dutch landrace** | 8482 | 27.77 (1,2) | 0.034 | 0.933 | -4744 | 5.943 (1,2) | 0.135 | 0.748 | -10310 | 30.58 (1,2) | 0.031 | 0.939 |
| **Great Yorkshire** | 7063 | 23.57 (1,2) | 0.040 | 0.922 | -4718 | 6.375 (1,2) | 0.128 | 0.761 | 7063 | 23.57 (1,2) | 0.040 | 0.922 |
| **Haldanes** |  |  |  |  |  |  |  |  |  |  |  |  |
| **Dutch landrace** | 0.032 | 27.77 (1,2) | 0.034 | 0.933 | -0.030 | 5.943 (1,2) | 0.135 | 0.748 | -0.032 | 30.58 (1,2) | 0.031 | 0.939 |
| **Great Yorkshire** | 0.032 | 23.59 (1,2) | 0.040 | 0.922 | -0.029 | 6.377 (1,2) | 0.128 | 0.761 | 0.032 | 23.59 (1,2) | 0.040 | 0.922 |

**Additional Reference**

1. Merks JWM. One century of genetic changes in pigs and the future needs. BSAS Occas. Publ. 2000;8–19.
